# Supplementary material for: Effects of cochlear implantation on gait performance in adults with hearing impairment: A systematic review
Source: PLoS One. 2025 Feb 28;20(2):e0319322. doi: 10.1371/journal.pone.0319322 (PMC11870346; doi:10.1371/journal.pone.0319322)
Supplement: S3 — (DOCX) [file pone.0319322.s003.docx]

S3. Supporting information for quality assessment in PEDro scale

|  | **Buhl et al. (2018)** | **Kaczmarczyk et al. (2019)** | **Kluenter et al. (2009)** | **Le Nobel et al. (2016)** | **Shayman et al. (2017)** | **Stieger et al. (2018)** | **Weaver et al. (2017)** |
| --- | --- | --- | --- | --- | --- | --- | --- |
| Inclusion and source | Yes | Yes | Yes | Yes | No | No | Yes |
| Random allocation | No | No | No | No | No | No | No |
| Concealed allocation | No | No | No | No | No | No | No |
| Baseline comparability | Yes | Yes | No | Yes | Unclear | Yes | Yes |
| Blinded subjects | No | No | No | No | No | No | No |
| Blinded therapists | No | No | No | No | No | No | No |
| Blinded assessors | No | No | No | No | No | No | No |
| Outcomes for > 85% | Yes | Yes | Yes | No | Yes | Yes | Yes |
| Intention to treat analysis or all subjects received intervention | Yes | Yes | Yes | Yes | Yes | Yes | Yes |
| Between group comparisons | Yes | Yes | Yes | Yes | Yes | Yes | Yes |
| Mean and variability of data presented | Yes | Yes | Yes | Yes | Yes | Yes | Yes |
| **Total score** | **5** | **5** | **5** | **4** | **4** | **5** | **4** |
