# Supplementary material for: Effects of cochlear implantation on gait performance in adults with hearing impairment: A systematic review
Source: PLoS One. 2025 Feb 28;20(2):e0319322. doi: 10.1371/journal.pone.0319322 (PMC11870346; doi:10.1371/journal.pone.0319322)
Supplement: Attachment — Submitted filename: Response to Reviewers.pdf [file pone.0319322.s004.pdf]

## Point-by-Point Response to Reviewers

**Manuscript titled "Effects of cochlear implantation on gait performance in adults with hearing impairment: A systematic review"**

Dear editorial board and reviewers,

We would like to express our deep gratitude for taking the time and effort to review our manuscript, and for your contributions, which we are certain will benefit this review paper. Below are the responses to each of the reviewer's comments. In addition, changes were made in the text and marked in red font. We carefully addressed the minor comments and the major concerns of the reviewers. We hope that your efforts and ours will be worth publishing in PLOS ONE.

### Reviewer 1

**Comment 1:**

The studies in this systematic review address two separate research questions:

1) Does cochlear implantation improve gait? This can only be addressed by the prospective studies that measured gait before and after implantation. There are 5 such studies: Buhl (2018), Kaczmarczyk (2019), Kluenter (2009), Le Nobel (2016), Stieger (2018).

2) If you have a CI, is gait affected by whether the CI is on or off? This question is addressed by 3 studies: Shayman (2017), Weaver (2017), and also Le Nobel (2016) (which addresses both research questions).

I would like the authors of this systemic review to more explicitly distinguish between these two research questions, and summarize the corresponding evidence separately.
Research question 1 can inform the important clinical decision of whether or not to implant; CI surgery is expected to degrade residual acoustic hearing, and may have a negative impact on vestibular function.
Regarding research question 2, comparisons of gait with CI off and on may improve the understanding of the mechanisms behind the observed post-op gait improvement relative to pre-op.

**Response** 1:

Thank you for your thoughtful comment. We have revised the manuscript to clarify the differences between prospective and on-off methods.

We now explicitly add this data where examination was conducted with CI On or Off status in Table 1a and also in 1b.

This comment has been addressed in four other sections of the manuscript. First, we included the relevant changes in the Study Design subsection. Second, in the Gait Outcomes following CI subsection. Third we discussed this issue in the discussion section. Finally, we mentioned it in the future recommendations. Please see these relevant changes in:

Page 11, Lines 177-184. In the study design section: "*It is important to note that the included studies used different methodologies. One approach was a prospective study that involved both pre-intervention and post-intervention assessments. The other approach assessed gait under on-off conditions, meaning gait was evaluated once with the CI turned on and once with it turned off. Five studies conducted pre- and post-intervention assessments (42,43,45,49,50), two studies assessed gait*

*with the implant in both on and off statuses (44,46), and one study employed both pre- and post-implant assessments along with on-off evaluations (50)"*

Page 15, Lines 262-270. In the Gait Outcomes following CI subsection: "*In addition, it is important to highlight the improved outcomes of gait based on the methodological approaches introduced above that utilized prospective design or on-off conditions. By utilizing prospective methods, one study (42) indicated an improvement in gait performance three months post-implant gait stability ratio, step time, single-support phase, tandem walk speed, and CoM. In contrast, studies with follow-ups averaging between 37 days and two months reported either a decline or no change in outcomes after CI (43, 45, 49). Studies using the on-off design reported mixed outcomes. Two studies reported no difference between the implant being turned on and off (46, 50), while another study noted improved gait velocity, when implants were activated compared to the off state (44)".*

Pages 18 and 19, lines 360-373. In the discussion, Study design and implementation of on-off methods: "*This review highlights two key study designs: Prospective (42,43,45,49,50) and on-off designs, with hearing input is assessed with the CI turned on (on condition) and off (off condition) (44,46). One of these studies (50) employed a mixed-method approach, utilizing both prospective and on-off designs. In the prospective approach, evaluations were conducted before and after implantation to determine whether CIs improve gait. The review identified variations in follow-up duration and outcomes among these studies, with longer follow-up periods (up to three months) showing gait improvements. In contrast, the on-off approach did not demonstrate gait improvement when comparing CI-on and CI-off conditions. However, this approach may enhance our understanding of the mechanisms underlying post-implant gait improvements, which are thought to arise from neuroplastic changes in response to increased audibility (29). Combining these two approaches could provide a more comprehensive understanding of gait improvements induced by cochlear implantation and audibility enhancement. Nonetheless, longer durations of CI use may be necessary to observe significant and sustained outcomes.*"

Page 21, lines 421-424. In Recommendation for future research:" *The implementation of on-off evaluations should also be considered to better understand the mechanistic relationship between hearing and gait. Combining prospective and on-off designs, with post-implant on-off evaluations conducted beyond three months after implantation, could reveal significant outcomes and provide deeper insights into the mechanisms underlying post-CI improvements".*

We believe that the manuscript has been strengthened by these changes.

**Comment 2:**

A minor point is that although the authors here say that Shayman (2017) had N = 3, only one participant had cochlear implants, so it is actually N = 1 for the purpose of this systematic review.

**Response 2**:

Thank you for your comment. We have revised this in Table 1a and also explicitly stated this on page 10, in line 165 "*one (out of three participants)*", and on page 19, in line 375 "*one participant*".

**Reviewer 2**

**Comment 1:**

Thank you for reviewing this study, which presents an interesting topic for the field of audiology and motor control.
I congratulate the authors on the study; however, I felt that a more robust and evidence-based justification was needed as to why these individuals' gait improved after CI.
I suggest that the authors write one or two paragraphs showing that the same thing happens in children with hearing loss who underwent CI surgery. The evidence shows that hearing again improves these individuals' balance, and with better balance, other motor skills that depend on balance to be performed, such as walking, can improve, both in deaf children with lesser degrees of hearing loss and in those who use CI. Below are the suggested references for the authors to write the requested paragraphs.

Cushing SL, Pothier D, Hughes C, Hubbard BJ, Gordon KA, Papsin BC. Providing auditory cues to improve stability in children who are deaf. Laryngoscope. 2012; 122: 101-2. Doi: 10.1002/lary.23807.

De Kegel A, Maes L, Baetens T, Dhooge I, Van Waelvelde H. The influence of a vestibular dysfunction on the motor development of hearing-impaired children. Laryngoscope. 2012; 122: 2837-43. Doi: 10.1002/lary.23529.

Hamzehpour F, Absalan A, Pirasteh E, Sharafi Z, Arbabsarjoo H. Investigating the effect of hearing aid use on the balance status of children with severe to profound congenital hearing loss using the pediatric clinical test of sensory interaction for balance. J Am Acad Audiol. 2021; 32: 303-7. Doi: 10.1055/s-0041-1728754.

Mazaheryazdi M, Moossavi A, Sarrafzadah J, Talebian S, Jalaie S. Study of the effects of hearing on static and dynamic postural function in children using cochlear implants. Int J Pediatr Otorhinolaryngol. 2017; 100: 18-22. Doi: 10.1016/j.ijporl.2017.06.002.

Melo RS, Lemos A, Macky CFST, Raposo MCF, Ferraz KM. Postural control assessment in students with normal hearing and sensorineural hearing loss. Braz J Otorhinolaryngol. 2015; 81: 431-8. Doi: 10.1016/j.bjorl.2014.08.014. Doi: 10.1016/j.bjorl.2014.08.014.

Melo RS, Lemos A, Raposo MCF, Belian RB, Ferraz KM. Balance performance of children and adolescents with sensorineural hearing loss: Repercussions of hearing loss degrees and etiological factors. Int J Pediatr Otorhinolaryngol. 2018; 110: 16-21. Doi: 10.1016/j.ijporl.2018.04.016.

Melo RS, Lemos A, Raposo MCF, Monteiro MG, Lambertz D, Ferraz KM. Repercussions of the hearing loss degrees and vestibular dysfunction on the static balance of children with sensorineural hearing loss. Phys Ther. 2021; 101: pzab177. Doi: 10.1093/ptj/pzab177.

Melo RS, Lemos A, Wiesiolek CC, Soares LGM, Raposo MCF, Lambertz D, et al. Postural sway velocity of deaf children with and without vestibular dysfunction. Sensors (Basel). 2024; 24: 3888. Doi: 10.3390/s24123888.

Suarez H, Alonso R, Arocena S, Ferreira E, Roman CS, Suarez A, et al. Sensorimotor interaction in deaf children. Relationship between gait performance and hearing input during childhood assessed in pre-lingual cochlear implant users. Acta Otolaryngol. 2017; 137: 346-51. Doi: 10.1080/00016489.2016.1247496.

Wolter NE, Gordon KA, Campos J, Madrigal LDV, Papsin BC, Cushing SL. Impact of the sensory environment on balance in children with bilateral cochleovestibular loss. Hear Res. 2021; 400: 108134. Doi: 10.1016/j.heares.2020.108134.

Zarei H, Norasteh AA, King L. The effect of auditory cues on static postural control: a systematic review and meta-analysis. Audiol Neurotol. 2022; 27: 427-36. Doi: 10.1159/000525951.

**Response 1**:

Thank you for your comment and your feedback. The addition of the reviewer's suggestion is indeed insightful for the current review. We added two paragraphs in the introduction section to address this and we of course used the references highlighted in red above that the reviewer has suggested. Thank you very much for this effort.

Please see these new paragraphs in the introduction section, page 4, lines 88-95 "*In line with this, previous research also shows that children with sensorineural hearing loss face significant challenges in balance, showing greater postural sway and instability (34–36), which is further affected by the severity of hearing loss (37), affecting both static postural control and dynamic balance during activities like walking, where sensory input integration is essential (3)* .

*Interventions using auditory stimulations, especially CIs, have shown a potential to improve postural stability and balance, reduce postural sway, and positively affect dynamic motor skills like gait in children (38–41).*

We are sincerely grateful to the reviewers' comments, we believe that the manuscript has been strengthened by these changes.
